# Analysis of emerging organic contaminants in water, fish and suspended particulate matter (SPM) in the Joint Danube Survey using solid-phase extraction followed by UHPLC-MS-MS and GC–MS analysis

**DOI:** 10.1016/j.scitotenv.2017.07.039

**Published:** 2017-12-31

**Authors:** Robert Loos, Simona Tavazzi, Giulio Mariani, Gert Suurkuusk, Bruno Paracchini, Gunther Umlauf

**Affiliations:** European Commission, Joint Research Centre (JRC), Directorate D – Sustainable Resources, Water and Marine Resources, I-21027 Ispra, VA, Italy

**Keywords:** Danube River, Water monitoring, UHPLC-MS-MS, GC-MS, Water Framework Directive, Emerging organic contaminants

## Abstract

In the third Joint Danube Survey (JDS3), emerging organic contaminants were analysed in the dissolved water phase of samples from the Danube River and its major tributaries. Analyses were performed using solid-phase extraction (SPE) followed by ultra-high-pressure liquid chromatography triple-quadrupole mass spectrometry (UHPLC-MS-MS) and gas chromatography–mass spectrometry (GC–MS). The polar organic compounds analysed by UHPLC-MS-MS were 1H-benzotriazole, methylbenzotriazoles, carbamazepine, 10,11-dihydro-10,11-dihydroxy-carbamazepine, diclofenac, sulfamethox-azole, 2,4-D (2,4-dichlorophenoxyacetic acid), MCPA (2-methyl-4-chlorophenoxyacetic acid), metolachlor, cybutryne (irgarol), terbutryn, DEET (*N*,*N*-diethyl-*m*-toluamide), and several perfluoroalkyl acids (C_6_–C_9_; C_8_ = perfluorooctanoic acid (PFOA)) and perfluorooctansulfonic acid (PFOS). In addition, several organophosphorus flame retardants were analysed by GC-MS. The most relevant compounds identified in the 71 water samples, in terms of highest median and maximum concentrations, were 1H-benzotriazole, tris(1-chloro-2-propyl)phosphate (TCPP), methylbenzotriazoles, carbama-zepine and its metabolite, DEET, sulfamethoxazole, tris(isobutyl)phosphate (TiBP), tris(2-chloroethyl)phosphate (TCEP), PFOA, PFOS and diclofenac. The concentrations of these compounds in the samples were generally below the environmental quality standard (EQS) threshold values, with the exception of PFOS, the concentration of which exceeded the annual average water EQS limit of 0.65 ng/L along the whole river, and also exceeded the fish biota EQS of 9.1 μg/kg. In addition, the proposed EQS for diclofenac, of 0.1 μg/L, was exceeded in the Arges River in Romania (255 ng/L).

## Highlights

• 71 water samples from the Danube River and its tributaries were analysed.
• Most relevant micropollutants were benzotriazoles, pharmaceuticals, organophosphorus compounds, and PFOS/A.
• PFOS concentrations exceed its environmental quality standard (EQS).
• Concentrations and loads are similar for the years 2007 and 2013.

## Graphical abstract

**Figure:**
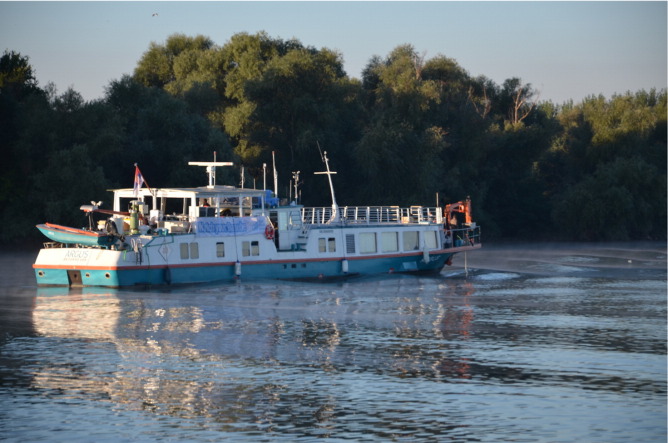

## 1 Introduction

After the first two Joint Danube Surveys (JDS) in 2001 and 2007 (ttp://www.icpdr.org/jds/), JDS3, organised by the International Commission for the Protection of the Danube River (ICPDR), was undertaken from 13 August to 25 September 2013 along the Danube River in nine countries from Germany to the Danube Delta in Eastern Europe. Three boats sampled 68 sites along a 2581-km stretch of the river; 14 of the sites were located in the mouths of tributaries or side arms (Slobodnik, 2015; http://www.danubesurvey.org).

The findings of the second survey (JDS2) confirmed the previous conclusions of the ICPDR that water quality in the main Danube River is generally improving. However, it also revealed specific problems, especially at a number of tributaries and downstream of large cities. In addition, it identified a number of specific areas of pollution with substances identified as a priority in the European Water Framework Directive (WFD) (EU (European Union), 2000, EU (European Union), 2008, EU (European Union), 2013), as well as with newly emerging contaminants, necessitating further, more extensive, examination, particularly in some tributaries (Liška, 2015). For example, persistent hydrophobic alkylphenolic compounds (nonylphenol, nonylphenol monoethoxylate (NP_1_EO), nonylphenol diethoxylate (NP_2_EO) and octylphenol) were found to be accumulating in the Danube sediments, with maximum concentrations in the mg/kg range (Micić and Hofmann, 2009).

For JDS2 our laboratory analysed 34 selected polar organic contaminants in water from the Danube River and its main tributaries (in the dissolved water phase). At this time, the focus was on pharmaceutical compounds (bezafibrate, carbamazepine, diclofenac, gemfibrozil, ibuprofen, ketoprofen, naproxen, sulfamethoxazole), pesticides and their degradation products (atrazine, bentazone, 2,4-D, desethylatrazine, desethylterbutylazine, diuron, isoproturon, mecoprop, simazine, terbutylazine), perfluorinated acids (perfluoro-octanoic acid (PFOA) and perfluorooctansulfonic acid (PFOS)) and endocrine-disrupting compounds, such as nonylphenol, octylphenol, nonylphenoxy acetic acid (NPE_1_C), bisphenol A, oestrone, oestradiol and caffeine. The most relevant compounds identified in the Danube River Basin in the year 2007 in terms of frequency of detection and concentration levels were caffeine (median concentration 87 ng/L), NPE_1_C (49 ng/L), carbamazepine (33 ng/L), PFOA (17 ng/L), sulfamethoxazole (16 ng/L), desethylatrazine (11 ng/L) and 2,4-D (10 ng/L). The highest contamination levels were found in the area around Budapest and in some tributary rivers, namely the Arges (Romania), the Timok (Bulgaria), the Rusenski Lom (Bulgaria) and the Velika Morava (Serbia) (Loos et al., 2010).

Relatively little information on the contamination of the Danube River or its tributaries by organic chemical substances is available in the public domain (Loos et al., 2010). However, since JDS2 in 2007, research findings have been published regarding contamination of the Danube in Serbia with pharmaceuticals (Radović et al., 2012), constituents of personal care or home cleaning products (Milic et al., 2014), caffeine (Grujić Letić et al., 2015) and pesticides (Antić et al., 2015).

JDS3 was an international longitudinal survey undertaken with the aim of obtaining comparable and reliable information on water quality throughout the length of the Danube River, including its major tributaries, to overcome the current information gaps and enable the implementation of the WFD (EU (European Union), 2000, Liška, 2015). In this sense, the objective of our work was the analysis of selected “emerging” organic chemical pollutants including some new priority substances of the WFD (cybutryne; terbutryn; PFOS), and to compare the contamination levels for some of the substances in a trend analysis with the results of JDS2 of the year 2007.

## 2 Materials and methods

### 2.1 Description of the Danube River

The Danube is the longest river in the European Union and Europe's second longest river after the Volga. Its catchment area covers 801,500 km^2^, and is home to approximately 81 million inhabitants in 19 countries. From the Black Forest in Germany it flows eastwards for some 2780 km, passing through several Central and Eastern European capitals, before emptying into the Black Sea via the Danube Delta in Romania and Ukraine. In terms of hydrology, the Alps and the Carpathian mountains contribute high precipitation, while the Inn River in Austria contributes a discharge (735 m^3^/s) that is higher than the Danube flow itself at the confluence of the two rivers. The other major tributaries, the Sava River (1564 m^3^/s), the Tisza River (794 m^3^/s) and the Drava River (577 m^3^/s), contribute roughly half of the total discharge at the mouth of the river (Kirschner et al., 2009; http://www.danubesurvey.org/).

### 2.2 Sampling

JDS3 was undertaken from 13 August to 25 September 2013 along the Danube River. Three boats took samples at 68 sites along a 2581-km stretch of the river; 14 of the sites were in the mouths of tributaries or side arms. In addition to the 68 official JDS samples (Slobodnik, 2015), three additional samples—JDS 51a (between the Iskar and Olt tributaries), JDS 51b (Olt River) and JDS 63a (between the Siret and Prut tributaries)—were analysed, so that in total 71 water samples were investigated in this study. The investigated tributary rivers were the Morava (Austria/Slovakia), the Vah (Slovakia), the Drava (Croatia), the Tisa (Serbia), the Sava (Serbia), the Velika Morava (Serbia), the Timok (Serbia/Bulgaria), the Iskar (Bulgaria), the Olt (Romania), the Jantra (Bulgaria), the Russenski Lom (Bulgaria), the Arges (Romania), the Siret (Romania) and the Prut (Romania/Moldavia). The water samples for chemical analyses were grab samples taken (below the water surface) in the middle of the Danube River and its tributaries. Suspended particulate matter (SPM) samples were collected by the on-board centrifuge at the sampling site; however, in some cases, owing to time constraints, a stretch of the river between two sampling sites had to be sampled instead. A list of all sampling locations and sampling dates can be found in Slobodnik (2015), and the JDS3 overview map is available at http://www.danubesurvey.org/results.

For the organic contaminants analysed in this study, methanol pre-cleaned 1 L aluminum bottles (Scientifica Panzeri, Milan, Italy) were used for sample storage and transportation to the JRC. The samples, cooled with freezing elements, were shipped in Styropor boxes, and generally arrived at the JRC in Ispra (Italy) 2 days later, where they were stored in a fridge at ~ 4 °C.

### 2.3 Selection of the target compounds

The ICPDR identified the organic emerging substances (depicted in Table SI1 with their CAS no., log K_OW_ and description/use) to be analysed in JDS3. Cybutryne (a biocide, also known as irgarol, which is mainly used as an antifouling agent in paints for boats and vessels and which is applied at marine as well as at inland freshwater sites), terbutryn (a herbicide and biocide or algicide used in paints) and PFOS are new priority substances under the WFD (Directive 2013/39/EU; EU, 2013). Diclofenac, a non-steroidal anti-inflammatory drug used to treat pain, was proposed as a new priority substance in 2012 (with a proposed EQS of 0.1 μg/L; note that the Swiss Ecotox Centre proposes a lower PNEC of 0.05 μg/L; see Section 2.10), and is monitored under the newly introduced WFD ‘watch list’ mechanism (EU, 2013). Carbamazepine, a mood-stabilising drug used for the treatment of epilepsy, bipolar depression, excited psychosis, mania, and sleeping disorders, is one of the pharmaceuticals most often analysed in the environment, and 10,11-dihydro-10,11-dihydroxy-carbamazepine (CBZ-diOH) an important degradation product of carbamazepine for which only few monitoring data are available (De Laurentiis et al., 2012, Fenet et al., 2012, Hummel et al., 2006, Miao and Metcalfe, 2003). In addition, the sulfonamide antibiotic sulfamethoxazole, used to treat urinary tract infections, sinusitis and toxoplasmosis, is often detected in the aqueous environment (Johnson et al., 2015, Straub, 2016) and, along with the herbicides 2,4-D, MCPA and metolachlor (because frequently detected; Schreiner et al., 2016) and the insect repellent DEET (Costanzo et al., 2007), was included in the study. Benzotriazoles (1H-benzotriazole and 5-methyl-1H-benzotriazole) were chosen because they are produced in high volume (being used as corrosion inhibitors) and detected in industrial and urban areas (Weiss et al., 2006) but were analysed in only selected samples in JDS2 (Loos et al., 2010). Note that 4- and 5-methyl-1H-benzotriazole cannot be separated chromatographically and, therefore, only the total concentration of methylbenzotriazoles is given in the results. In addition, several perfluoroalkyl substances were analysed because PFOA was one of the most important contaminants in JDS2, originating mostly from a fluoropolymer production plant located in Germany on the Inn River tributary (Hangen et al., 2010). Carbamazepine, diclofenac, sulfamethoxazole, 2, 4-D, PFOS and PFOA were analysed in JDS2. In addition, several organophosphorus compounds (OPCs) mainly used as flame retardants and plasticisers were analysed by GC–MS (Andresen et al., 2004, Martinez-Carballo et al., 2007, Regnery and Püttmann, 2010, Reemtsma et al., 2008, Van der Veen and de Boer, 2012, Wolschke et al., 2015).

### 2.4 Solid-phase extraction (SPE)

The water samples were extracted by automated solid-phase extraction (SPE) with Oasis HLB (200 mg) cartridges (Waters Corporation, Milford, MA, USA) using an Autotrace AT280 SPE workstation (Thermo Scientific, Waltham, MA, USA). The extraction volume was 1 L and the water was not filtered, but decanted by pouring the water after sedimentation slowly from the sample bottles into clean 1-L glass (Schott Duran®) bottles. Before extraction, the samples were spiked with the internal surrogate standards (depicted in Table SI2) used for ‘isotope dilution’ quantification. Two 50-μL aliquots of labelled internal standard (IS) solution (1 mg/L for the polar UHPLC-MS-MS compounds and 2 mg/L for the OPCs) were added to the 1-L water samples so that the spiking level in the water samples was between 50 and 100 ng/L; the water samples were then shaken for 10 s. The ISs were provided by Chiron AS (Trondheim, Norway), CDN Isotopes (Pointe-Claire, Canada), Cambridge Isotope Labs (Andover, MA, USA), Hayashi Pure Chemical Ind. Co. (Osaka, Japan), Dr. Ehrenstorfer (Augsburg, Germany) and Wellington Labs (Guelph, Canada).

The objective of our developed SPE procedure was to extract only 1 L water with one SPE cartridge and use the extract for both UHPLC-MS-MS and GC–MS analysis. The Oasis HLB (200 mg) cartridges were conditioned with 10 mL of ethyl acetate, 10 mL of methanol and 10 mL of Milli-Q water. The 1-L water samples were then loaded on the cartridges at a flow rate of 10 mL/min. After drying the cartridges with nitrogen for 30 min, the samples were eluted with 10 mL of ethyl acetate at 5 mL/min. Half of the resulting extract (i.e. about 5 mL) was used for the UHPLC-MS-MS analysis. The HLB column was then eluted again with 10 mL methanol containing 0.1% ammonia in order to increase the recovery of the polar compounds (including PFAS). The combined ethyl acetate / methanol extract was then evaporated to dryness using a TurboVap® system (Caliper Life Sciences) and then reconstituted in 0.2 mL of mobile phase A for UHPLC-MS-MS analysis. The remaining ethyl acetate aliquot (i.e. about 5 mL) was evaporated to 50–100 μL under nitrogen flow for GC–MS determination of the OPCs. Ethyl acetate was selected for the SPE due to its GC–MS applicability (it can be injected into a standard GC–MS column).

Recoveries were evaluated by extracting and analysing, in triplicate, 1-L Milli-Q water samples that had been spiked before extraction with native analytes; ISs were then added to the extracts for quantification. The spiking level was 10 ng/L for each analyte. The ratios of analyte to ISs were evaluated and compared with the same ratios obtained by analysing a standard solution containing the same concentrations of native compounds and labelled ones. The recovery results are depicted in Table SI3.

### 2.5 Stability studies

Unfortunately, the water samples could not be extracted (and analysed) immediately after arrival at the JRC. On average, they were extracted after 68 days' storage in the fridge (range 27–106 days; 30 samples were stored for longer than 70 days). Therefore, in order to determine sample stability (storage at 4 °C in aluminum containers), three exemplary water samples (JDS11, JDS12, JDS16) were re-extracted and re-analysed after 173 days' storage. The ‘118 day variation’ (in %) between the two analyses (the first extraction was done after 55 days) is shown in Table SI4.

These results show that most substances tested (1H-benzotriazole, 2,4-D, carbamazepine and its metabolite, MCPA, metolachlor, sulfamethoxazole, terbutryn, PFHxA, PFOA and PFOS) were relatively stable under the storage conditions. The increase in the concentration of methylbenzotriazoles cannot be explained. Less stable substances were DEET, diclofenac and cybutryne. Perfluorinated substances are usually ‘persistent’ compounds so the reason for the decrease in the concentration of PFBS, PFHpA and PFNA is not clear.

In general, however, the monitoring data for individual samples stored for 55 or 100 days are relatively uniform, and no major differences can be observed, except in the case of cybutryne, the concentration of which is clearly lower in the samples that had been stored longer.

### 2.6 Extraction of fish and SPM for PFOS analyses

Extraction of freeze-dried fish liver and SPM samples (ca. 1 g) was performed by a modified Powley method (Berger et al., 2009, Powley et al., 2005), which extracts perfluoroalkyl substances (PFAS) from solid matrices such as fish by ultra-sonication with methanol or acetonitrile. Extraction was performed after addition of ^13^C_4_-PFOS IS by repeated ultrasonic extraction with methanol followed by ENVI-Carb sorbent clean-up as described in Roland et al. (2014). Fish and SPM data from 2007 (JDS2) were obtained from frozen samples stored at the JRC and analysed together with the samples from 2013 (JDS3). Extraction efficiency was evaluated by subsequent methanol extraction of a selected SPM and fish liver sample. Each sample was re-extracted four times and the sum of concentrations from the first three extraction steps was calculated as percentage of the total amount of PFOS extracted, giving an extraction efficiency of 99% for SPM and 94% for liver.

### 2.7 UHPLC-MS-MS analysis and quality assurance/quality control

Analyses were performed by ultra-high-pressure liquid chromatography tandem mass spectrometry (UHPLC-MS-MS), which was performed with an Acquity® UHPLC system (Waters Corporation) coupled to a hybrid triple-quadrupole linear ion trap mass spectrometer 5500 QTRAP® with a turbo ion spray source from AB SCIEX (Foster City, CA, USA). The analytical column used was an Acquity® UPLC® BEH C_18_, 1.7 μm, 50 × 2.1 mm column (Waters Corporation); the flow rate was 600 μL/min and the injection volume 5 μL. Mobile phases used were (A) water–methanol (95:5%, v/v) containing 0.1% acetic acid, and (B) acetonitrile–methanol (50:50%, v/v) containing 0.1% acetic acid. Chromatography was performed in gradient mode, starting with 90% A, which was held for 1 min, followed by gradient rise to 90% B over 6 min and, finally, re-equilibration, giving a total analysis time of 10 min. Table SI5 gives the analytical UHPLC-MS-MS conditions (e.g. retention times; MS-MS transitions) in detail; note that the compounds determined in negative ionization mode have negative potentials (DP; EP; CE).

The compounds present in samples were identified by matching their retention times and their specific MS-MS multiple reaction monitoring (MRM) transitions. Quantification was performed by isotope dilution using deuterated or ^13^C-labelled ISs, as shown in Table SI2. All calculations were based on the ratios of the chromatographic peak area for the MRM precursor–product ion transitions for analytes to those for the ISs. The relative response factors of the compounds in relation to the ISs were calculated in all cases. Thus, the reported concentrations are corrected for the recoveries of the compounds.

The analytical method was fully validated including calculation of linearity, trueness, repeatability, day-to-day variation, and intermediate precision. The linearity of the full SPE UHPLC-MS-MS method was studied at a real water concentration range of 0.2–100 ng/L with 1-L Milli-Q water blank samples (results given in Table SI6; all R^2^ value were > 0.986). Repeatability, day-to-day variation, and intermediate precision were estimated with one-way ANOVA (results given in Table SI7; the RSD was only in one case > 30%). In addition, a trueness significance *t*-test was performed (results not shown). The limits of detection (LODs) and limits of quantification (LOQs) for the SPE-UHPLC-MS-MS procedure were estimated (Magnusson and Örnemark, 2014) from the mean concentration of blank water samples (50 mL Milli-Q water) plus three times the standard deviation (10 times the standard deviation in the case of the LOQs). Ion suppression and matrix effects of the samples were not checked; they are relatively low for river water samples. The results of the LOD and LOQ estimation for the target analytes are shown in Table SI8. In addition, several field blanks were included in the campaign.

### 2.8 GC-MS analysis of organophosphorus compounds (OPCs)

An Agilent 6890 N GC system with a RXI-17SIL MS 60 m column coupled to an Agilent 5973 Mass Selective Detector were used. For detailed technical information, see Table SI9. The GC–MS SIM parameters for the OPCs are given in Table SI10. Limits of detection were visually estimated in the chromatograms of blank samples as the lowest signals that gave a signal-to-noise ratio of about 5:1 for LOD and 1:1 for LOQ. The results are shown in Table SI8.

The analytes were identified in GC–MS SIM mode by recording the target ion and one qualifier ion. The target analytes were identified by retention time comparison with the corresponding standards and the isotopic ratio between the two ions recorded (± 20%). For the OPC analysis, linearity was studied for the extraction and GC–MS analysis of real (Milli-Q) water samples in a concentration range of 10–500 ng/L on 8 different days. The average R^2^ values, given in Table SI6–2 were in all cases > 0.970. Repeatability was studied by the replicate analysis of control samples at two spiked concentration levels (30 and 300 ng/L), and the RSDs were in all cases < 21% (Table SI7–2).

### 2.9 Statistical analyses

Frequency of positive detection (freq, %), as well as the average, median (med) and 90th percentile values (P90), were quantified using Excel (Microsoft Corporation, Seattle, WA, USA). Non-detect values were set to half the LOQ for these calculations (in accordance with Directive 2009/90/EC) (Helsel, 2006).

### 2.10 Environmental quality standards

The EQS were selected from Directive 2013/39/EU (EU, 2013), the Swiss Ecotox Centre (http://www.ecotoxcentre.ch/expert-service/quality-standards/), the German on-line Information System on Ecotoxicology and Environmental Quality Targets (UBA) (https://webetox.uba.de/webETOX/public/search/ziel.do), and for the perfluoroalkyl substances from the substance dossiers of the Italian EQS working group (Valsecchi et al., 2017).

## 3 Results and discussion

### 3.1 Chemical compounds identified

Table 1 summarises the analytical results for the organic contaminants that were measured in the dissolved water phase of the Danube River (55 samples) and its tributaries (16 samples) during JDS3 in August–September 2013. The minimum, median, average, 90th percentile and maximum concentrations are given.

**Table 1:** Monitoring results for emerging organic contaminants in the dissolved water phase of the Danube River and tributaries Number of samples = 71; unit: ng/L; the results < LOQ were replaced by zero, and in case of cybutryne, PFHpA, EHDP, TMPP, T35DMPP, and TnPP due to the low detection frequency (D.F.) by LOQ/2; P90 = 90th percentile; EQS = environmental quality standard (annual average).

Analyte	D.F. (%)	Min.	Median	Average	P90	Max.	LOQ	EQS
1-H-Benzotriazole	100	1.5	260	287	462	1550	0.66	19,000
5-Methyl-1H-benzotriazole	100	9.5	57	67	115	290	0.53	20,000
Carbamazepine (CBZ)	100	3.6	25	26	36	68	0.15	2000
10,11-Dihydro-10,11-dihydroxy-carbamazepine	100	13	43	53	86	161	0.30	100,000
Diclofenac	75	< LOQ	3.6	10	15	255	0.86	100
Sulfamethoxazole	100	3.7	18	23	40	141	0.10	600
2,4-D (2,4-dichlorophenoxyacetic acid)	96	< LOQ	1.7	2.9	4.8	22	0.22	200
MCPA (2-methyl-4-chlorophenoxyacetic acid)	93	< LOQ	1.7	2.2	4.0	12	0.15	660
Metolachlor	99	< LOQ	5.4	6.3	9.0	39	1.73	200
Cybutryne (irgarol)	24	< LOQ	0.09	0.11	0.5	0.83	0.18	2.5
Terbutryn	96	< LOQ	2.9	3.1	4.4	11	0.64	65
DEET (N,N-diethyl-m-toluamide)	100	< LOQ	10	13	23	81	1.93	88,000
PFBS (perfluorobutane sulfonic acid)	94	< LOQ	1.4	1.6	2.6	3.7	0.55	3000
PFHxA (perfluorohexanoic acid)	92	< LOQ	4.0	4.0	6.7	8.5	1.10	1000
PFHpA (perfluoroheptanoic acid)	38	< LOQ	1.6	3.4	6.6	19	3.20	n.a.
PFOA (perfluorooctanoic acid)	100	< LOQ	4.9	8.1	18	37	1.07	100
PFNA (perfluorononanoic acid)	79	< LOQ	1.1	1.2	2.7	3.3	0.66	n.a.
PFOS (perfluorooctane sulfonic acid)	94	< LOQ	5.9	7.2	13	26	1.09	0.65
Tri-n-propyl phosphate (TnPP)	11	< LOQ	0.35	0.42	0.71	2.1	0.70	n.a.
Tris(isobutyl) phosphate (TiBP)	100	2.5	18	20	27	97	0.51	11,000
Tris(n-butyl) phosphate (TnBP)	99	0.4	4.2	5.6	7.2	70	0.30	10,000
Tris(1-chloro-2-propyl) phosphate (TCPP)	100	28	92	115	167	603	4.30	640,000
Tris(2-chloroethyl) phosphate (TCEP)	100	2.4	10	11	18	41	0.96	4000
Tris(2-butoxyethyl) phosphate (TBEP)	63	< LOQ	8.6	13	25	93	5.03	24,000
Tris(1,3-dichloropropyl) phosphate (TDCPP) (or 2,3)	99	< LOQ	11	11	16	28	2.83	10,000
Triphenyl phosphate (TPhP)	79	< LOQ	1.0	1.5	3.8	7.6	0.52	170
2-Ethylhexyl diphenyl phosphate (EHDP)	28	< LOQ	0.1	0.6	1.5	5.9	0.28	n.a.
Tris(methylphenyl) phosphate (TMPP)	17	< LOQ	0.40	1.3	2.2	13	0.79	n.a.
Tris(3,5-dimethylphenyl) phosphate (T35DMPP)	13	< LOQ	1.7	4.4	11	54	3.44	n.a.

#### 3.1.1 Detection frequencies

Overall, the detection frequencies for most compounds were very high (> 90%) (Table 1); only four substances were detected less frequently: cybutryne (24%; LOQ 0.18 ng/L), diclofenac (75%; LOQ 0.86 ng/L), PFHpA (38%; LOQ 3.20 ng/L) and PFNA (79%; LOQ 0.66 ng/L).

#### 3.1.2 Maximum concentrations

The compound detected at the highest concentrations was the corrosion inhibitor 1H-benzotriazole, with a median concentration of 260 ng/L, an average of 287 ng/L and a 90th percentile (P90) of 462 ng/L. The maximum concentration of 1H-benzotriazole, 1550 ng/L, was found in a sample taken from the Vah tributary (JDS18) in Slovakia, between Bratislava and Budapest. The second highest ranked substance was tris(1-chloro-2-propyl)phosphate (TCPP) with a median concentration of 92 ng/L and a maximum of 603 ng/L in the Timok River (JDS48). The concentration of methylbenzotriazoles was highest (290 ng/L) in the Arges tributary (JDS58) in Romania, close to the capital, Bucharest. Other substances detected at elevated concentrations at the same site were carbamazepine (max. 68 ng/L), 10,11-dihydro-10,11-dihydroxy-carbamazepine (CBZ-diOH) (max. 161 ng/L), sulfamethoxazole (max. 141 ng/L), tris(isobutyl)phosphate (TiBP) (max. 97 ng/L) and diclofenac (max. 255 ng/L), while at other sites there were high concentrations of DEET (max. 81 ng/L, in the Morava tributary, JDS12, between Austria and Slovakia, close to Bratislava), TCEP (max. 41 ng/L, in the Iskar River; JDS51), PFOA (max. 36.5 ng/L, in the Danube River downstream of Budapest; JDS22), PFOS (max. 26.2 ng/L, in the Danube River in Szob before Budapest; JDS20).

Usually smaller rivers or streams are more polluted than big rivers. In 2007, in samples taken for JDS2, the maximum concentrations of most chemicals were detected in tributary rivers rather than in the Danube itself (Loos et al., 2010). This trend was less pronounced in JDS3, which found that for only 7 out of 18 compounds (1H-benzotriazole, 2,4-D, DEET, diclofenac, MCPA, metolachlor, sulfamethoxazole) were maximum concentrations found in the tributary rivers.

### 3.2 Benzotriazoles

Benzotriazoles (1H-benzotriazole and tolyltriazole (4- and 5-methyl-1-H-benzotriazole)) are chemicals produced in high volumes that are mainly used as corrosion inhibitors in a variety of consumer products and industrial applications, and are widely distributed in the environment. They are mainly used as anti-corrosives in dishwasher detergents, industrial cooling systems, de-icing/anti-icing fluids (for aircraft), dry cleaning equipment, metal-cutting fluids, brake fluids, solid cooling lubricants and anti-freezing liquids. They are also used as flame inhibitors, ultraviolet light stabilisers in plastics and antifogging agents (Asimakopoulos et al., 2013, Herrero et al., 2013).

Benzotriazoles are weakly basic compounds of high polarity. The two common forms, benzotriazole and tolyltriazole (methylbenzotriazole), are soluble in water, relatively resistant to biodegradation, and are only partly removed by wastewater treatment (5-tolyltriazole, however, is readily biodegraded (Weiss et al., 2006). They are ubiquitous environmental contaminants, but have a limited biological activity; for example, acute toxicity to aquatic organisms is in the low to moderate mg/L range; their EQS are given in Table 1 (Weiss et al., 2006).

**Fig. 1:**
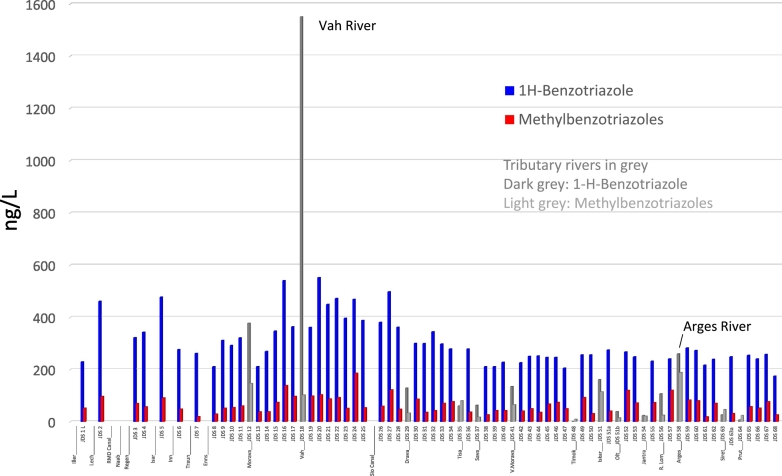
Benzotriazoles concentration profiles along the Danube River and tributaries.

In this study, the organic compound with the highest average concentration level, by far, was 1H-benzotriazole (287 ng/L), followed by TCPP (115 ng/L), the methyl-benzotriazoles (67 ng/L) and the carbamazepine metabolite CBZ-diOH (53 ng/L) (Table 1). However, examination of the individual results for 1H-benzotriazole shows that the concentration was lower than average in several downstream tributary samples, compared to the Danube itself. Fig. 1 presents the concentration profile for the benzotriazoles over the whole Danube River (including the tributaries), with higher concentrations (around 400–500 ng/L) found for 1H-benzotriazole in Germany, Austria and Slovakia, and around Budapest in Hungary, down to JDS32 (345 ng/L; Novi-Sad, Serbia), with the maximum found in the Vah River (1550 ng/L) in Slovakia.

The sampling sites with lower concentrations of 1H-benzotriazole are the downstream tributaries, and these are shown in grey in Fig. 1 (Drava 129 ng/L; Tisa 61 ng/L; Sava 63 ng/L; Velika Morava 135 ng/L; Timok 2 ng/L; Iskar 161 ng/L; Olt 39 ng/L; Jantra 24 ng/L; Russenski Lom 107 ng/L; Siret 27 ng/L; Prut 7 ng/L).

A similar trend was observed for the methylbenzotriazoles, for which lower concentrations were found than for 1H-benzotriazole (average 67 ng/L) (Fig. 1), although lower than average levels were observed in fewer tributaries (Drava 33 ng/L; Sava 18 ng/L; Timok 9 ng/L; Olt 15 ng/L; Jantra 21 ng/L; Russenski Lom 25 ng/L; Prut 24 ng/L). These results indicate that consumption of benzotriazoles is lower in the Eastern European countries of Serbia, Romania and Bulgaria than elsewhere in the Danube catchment area. A possible explanation for this could be that 1H-benzotriazole is used in dishwasher powder or tablets as a corrosion inhibitor but that dishwashers are less commonly used in the downstream countries.

### 3.3 Pharmaceuticals

The pharmaceuticals investigated in this study were carbamazepine, its metabolite 10,11-dihydro-10,11-dihydroxy-carbamazepine (CBZ-diOH), the antibiotic sulfamethoxazole and diclofenac. The highest concentrations were detected for the carbamazepine metabolite (maximum 161 ng/L, median 43 ng/L), followed by carbamazepine (maximum 68 ng/L, median 25 ng/L), sulfamethoxazole (maximum 141 ng/L, median 18 ng/L) and diclofenac (maximum 255 ng/L, median 3.6 ng/L).

#### 3.3.1 Carbamazepine and CBZ-diOH

**Fig. 2:**
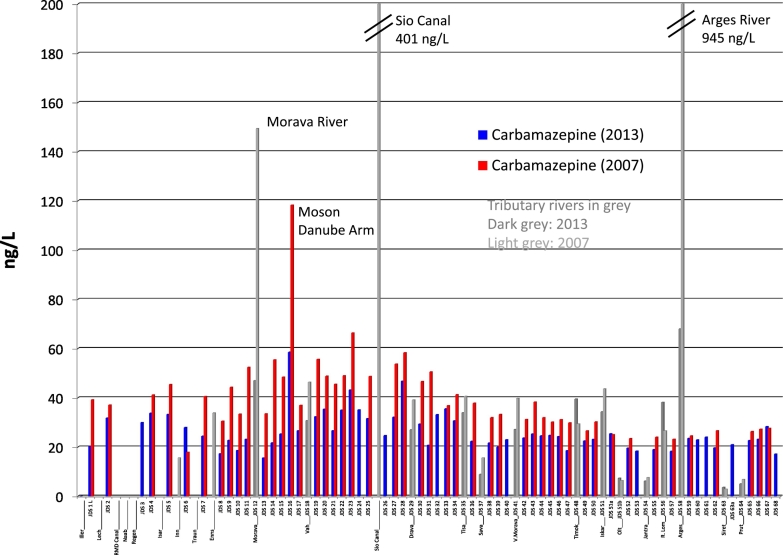
Carbamazepine concentration profile along the Danube River and tributaries for the years 2013 and 2007.

The analytical results for carbamazepine and its metabolite are in agreement with those of other studies, in which CBZ-diOH, the hydroxylated metabolite of carbamazepine, has been detected in waste and surface water at a higher concentration than the parent compound (De Laurentiis et al., 2012, Fenet et al., 2012, López-Serna et al., 2012, Hummel et al., 2006, Miao and Metcalfe, 2003). Levels of carbamazepine were slightly higher in the upstream part of the Danube than further downstream: around 30 ng/L in Germany and up to sample number JDS34 in Novi Sad, Serbia. After the Tisa tributary influent (JDS35), concentrations dropped to around 20 ng/L in Romania and Bulgaria, with slightly higher levels in some tributaries, and levels below 10 ng/L in the tributaries Olt, Jantra, Siret and Prut. The highest maximum concentrations were found in the Morava River (47 ng/L; JDS12) in Bratislava, in the Monson Danube Arm in Hungary (59 ng/L) downstream of Bratislava (JDS16), and in the Arges River in Romania (68 ng/L) (Fig. 2).

The comparison of the concentration profiles for carbamazepine along the Danube River for the years 2013 and 2007 (JDS2) shows that the concentrations in the upstream and middle parts of the river fell by approximately 20–30% (Fig. 2), which indicates improved wastewater treatment in the area of Budapest (Loos et al., 2010). It is less likely that consumption of carbamazepine was lower in 2013 than in 2007. In the lower part of the Danube River, however, the levels are quite similar in both years, which can be explained by the lower river flow and mass loads in 2013 as a result of ponding of the water at the Iron Gate Dam (JDS44) (see Section 3.9). In 2007 much higher concentrations were found as well in three tributaries (Morava, Sio Canal, Arges) (Fig. 2).

In addition, the ratio of CBZ to CBZ-diOH concentrations was calculated for all samples along the Danube River and a slight decreasing ratio from the source in Germany of approximately 0.7 to 0.5 in the lower part of the river was found which shows the degradation of carbamazepine in the river. A maximum of 1.7 was found in Szob (JDS20) upstream Budapest (see Figure SI1).

#### 3.3.2 Sulfamethoxazole

**Fig. 3:**
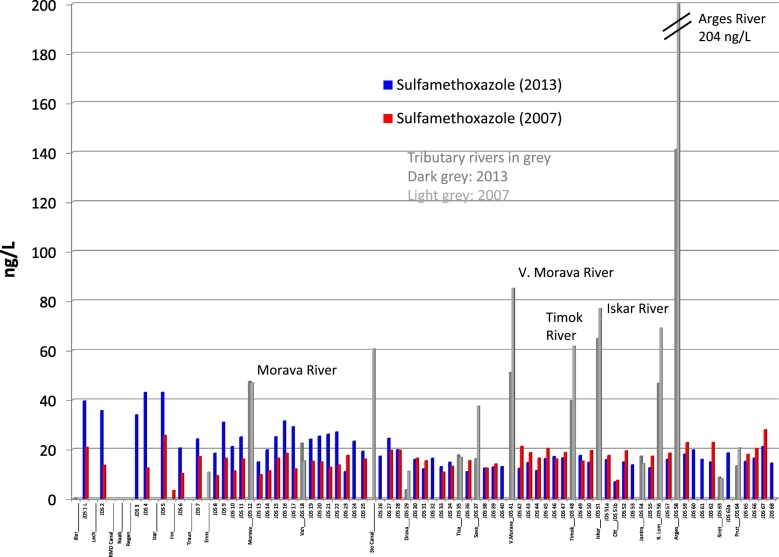
Sulfamethoxazole concentration profile along the Danube River and tributaries for the years 2013 and 2007.

The highest sulfamethoxazole concentrations were detected in the upper part of the Danube River in Germany (around 40 ng/L). The levels in the area from Bratislava to Budapest (JDS10–25) were slightly above 20 ng/L, and in the downstream part below 20 ng/L (around 15 ng/L). In contrast to carbamazepine, concentrations of sulfamethoxazole were higher in 2013 than in 2007 in the upper part of the Danube River (up to JDS27 in Hungary); in Germany the concentrations doubled to around 40 ng/L, which indicates an increased use of sulfamethoxazole in Germany (Fig. 3).

In the downstream part of the Danube, sulfamethoxazole levels were very similar in 2007 and 2013 (around 20 ng/L), and elevated levels were found in the tributaries Velika Morava (51 ng/L), Timok (40 ng/L), Iskar (65 ng/L), Russenski Lom (47 ng/L) and Arges (141 ng/L) (as for carbamazepine). In the downstream tributaries concentrations were higher in 2007 (Fig. 3).

These sulfamethoxazole concentrations are in good agreement to other monitoring studies (e.g. in the Thames River (UK; average 23 ng/L; max. 146 ng/L; Nakada et al., 2017). Straub (2016) collected > 5000 measured environmental concentrations for sulfamethoxazole in European surface waters, with a calculated median of 52 ng/L, which means that the concentrations in the Danube River are lower than the European median.

#### 3.3.3 Diclofenac

**Fig. 4:**
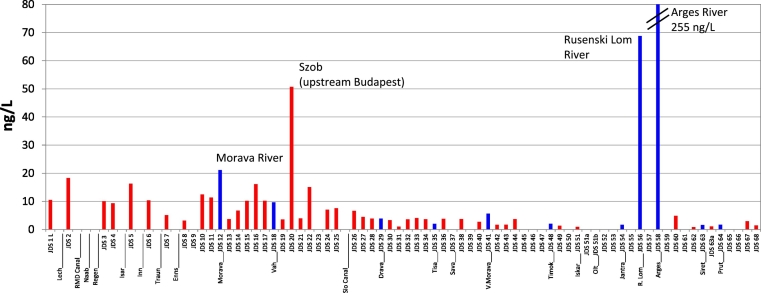
Diclofenac concentration profile along the Danube River and tributaries (year 2013). Tributaries in blue font.

Diclofenac concentrations were relatively low: between 10 and 20 ng/L in the upstream part of the Danube in Germany, around 10 ng/L between Vienna and Budapest, below 10 ng/L after Budapest, and reaching levels of only around 2 ng/L in the river mouth (Fig. 4). The highest diclofenac levels were found in Szob (Hungary; JDS20; upstream Budapest; 51 ng/L) and in the Rusenski Lom (69 ng/L), and the Arges (255 ng/L) tributaries in Romania. This result in the Arges River was confirmed by two other laboratories participating in JDS3 using analytical methods with higher detection limits: UBA (Vienna, Austria) reported a level of 0.24 μg/L and UFZ (Leipzig, Germany) a level of 0.32 μg/L, both exceeding the proposed diclofenac EQS of 0.1 μg/L (Deutsch and Sengl, 2015). The diclofenac levels are not compared with that from the year 2007 because of analytical problems for diclofenac in 2007.

Low diclofenac levels were found as well in the Ebro River (Spain) with a maximum of 260 ng/L in a tributary river of the Ebro (López-Serna et al., 2012). In the Thames River (UK) higher diclofenac concentrations were measured (average 38 ng/L; max. 330 ng/L) (Nakada et al., 2017).

### 3.4 Pesticides

In general, the polar herbicides analysed, 2,4-D, MCPA and metolachlor, are applied in agriculture during the main growing season from April to July. Thus, the low concentrations of these herbicides found in JDS samples taken in August and September are not representative, as was also found during the last surveys. In contrast, biocides used as antifouling agents in paints (cybutryne and terbutryn) are discharged on a regular basis. In some countries, terbutryn is used as both a herbicide and a biocide, resulting in continuous contamination of surface waters (Deutsch and Sengl, 2015).

The highest pesticide concentrations were found for the insect repellent DEET (median 10 ng/L, max. 81 ng/L), followed by metolachlor (median 5.4 ng/L, max. 39 ng/L), terbutryn (median 2.9 ng/L, max. 11 ng/L), 2,4-D (median 1.7 ng/L, max. 22 ng/L) and MCPA (median 1.7 ng/L, max. 12 ng/L). Cybutryne levels were very low (max. 0.83 ng/L; Table 1).

The concentration profile of DEET along the Danube River and its tributaries is shown in Figure SI2 in the supporting information. The highest concentrations (around 20 ng/L) were found in Germany and Hungary (Budapest). In the lower part of the Danube the levels decreased to around 10 ng/L, with maximum levels in the Morava (81 ng/L) and Arges (37 ng/L) Rivers.

DEET, metolachlor, terbutryn, MCPA and cybutryne were not analysed in 2007, so a comparison with 2007 is possible only for 2,4-D. In 2013, concentrations of 2,4-D were considerably lower than in 2007 (median 10 ng/L, P90 29 ng/L, max. 188 ng/L; see Figure SI3), which is an indication of reduced use of this herbicide. It should be however noted that exposure of surface waters to pesticides is heavily dependent on local conditions (e.g. pesticide application and land use) and therefore can be spatially and temporally variable.

DEET is a common environmental contaminant. In a review of the worldwide occurrence of DEET in the aquatic environment, concentrations ranging from 40 to 3000 ng/L in various water bodies were reported (Costanzo et al., 2007). Higher DEET concentrations were also found by Nakada et al. (2017) in the Thames River (UK) (average 95 ng/L; max. 590 ng/L). Similar metolachlor concentrations (average 5 ng/L; max. 13 ng/L) were reported from the Llobregat River (Spain; Köck-Schulmeyer et al., 2012), and the Ebro river basin (Spain; max. 5 ng/L; Ccanccapa et al., 2016). Very similar terbutryn levels were found as well in the Arade River estuary (Portugal; 2–5 ng/L; Gonzalez-Rey et al., 2015), the Ebro river basin (Spain; average 4 ng/L; max. 30 ng/L; Ccanccapa et al., 2016), and in Denmark (max. 14 ng/L; Vorkamp et al., 2014). Much higher terbutryn levels were found in small rivers in Germany (average 300 ng/L; Quednow and Püttmann, 2007). Cybutryn is usually found in harbours, marinas and coastal areas due to its use as an antifouling agent on ship and boat hulls (Vorkamp et al., 2014), which explains its low concentrations in the Danube River.

### 3.5 Perfluorinated acids

**Fig. 5:**
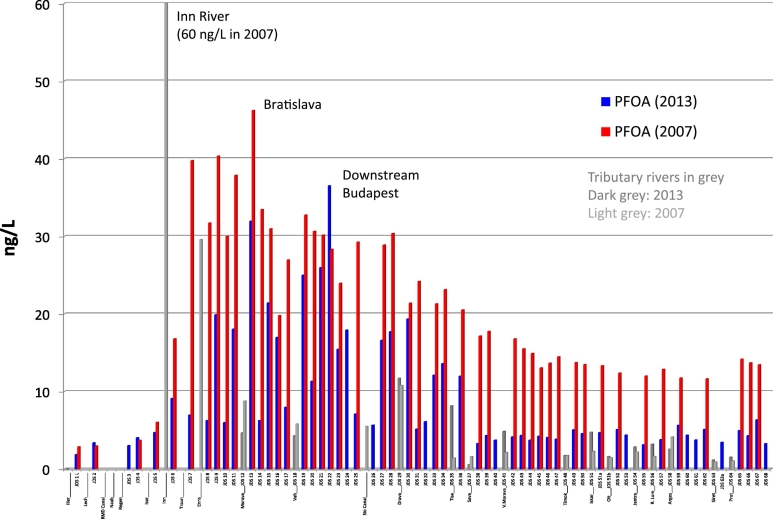
PFOA concentration profile along the Danube River and tributaries for the years 2013 and 2007.

In 2007, the Inn River (Germany/Austria) was the main source of PFOA contamination of the Danube River (Loos et al., 2010). Fig. 5 shows that this is no longer the case. In 2013, the highest PFOA levels, between 10 and 37 ng/L (median 4.9 ng/L), were detected in the Danube between JDS9 (Klosterneuburg, Austria) and JDS36 (downstream Novi Sad, Serbia); the highest concentration was found downstream Budapest. Discharge levels at the river mouth in Romania fell from 12 ng/L in 2007 to 5 ng/L in 2013 (Fig. 5).

**Fig. 6:**
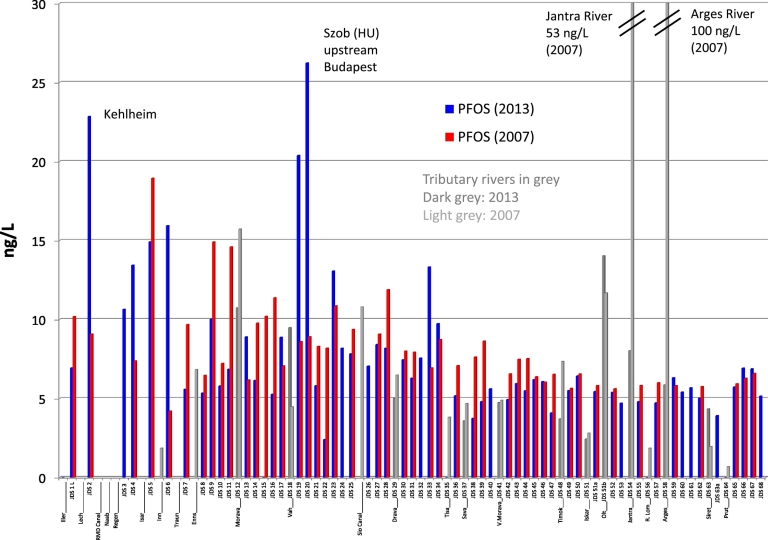
PFOS concentration profile along the Danube River and tributaries for the years 2013 and 2007.

In contrast, PFOS concentrations, which show a relatively uniform concentration profile, remained constant over the time period 2007–2013 (Fig. 6). In 2013, the highest PFOS concentrations were found at JDS2 (Kelheim, Germany; 23 ng/L) and at JDS19 and JDS20, before Budapest (Szob; 20 and 26 ng/L, respectively). PFOS levels in the Jantra and Arges tributaries decreased considerably from 2007 to 2013.

It is well known that PFOA is widely used as processing aid in the production of fluoropolymers (like PTFE) and discharged to the environment via waste water streams (Valsecchi et al., 2015). In fact, a fluoropolymer production plant is located in the Inn River basin in Germany/Austria. Our results indicate a decreased use of PFOA in this plant or better waste water treatment applied since 2007. PFOS, in contrast has more diffuse emission sources due to its widespread use in many industrial products such as inks, varnishes, waxes, fire-fighting foams, metal plating and cleaning, coating formulations, lubricants, water and oil repellents for leather, paper and textiles (Paul et al., 2009).

Similar but locally also higher PFAS concentrations have been reported in Italy (Valsecchi et al., 2015) and Spain (Campo et al., 2015).

### 3.6 Organophosphorus compounds

**Fig. 7:**
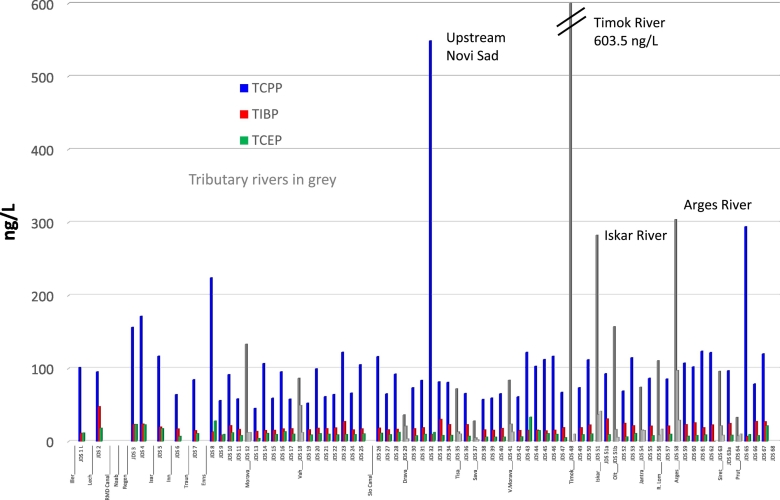
OPCs concentration profiles along the Danube River and tributaries for the year 2013.

Among the OPCs the highest concentrations found were of the flame-retardant compounds TCPP (median 92 ng/L, average 115 ng/L, maximum 603 ng/L), followed by TiBP (median 18 ng/L, maximum 97 ng/L), tris(2-butoxyethyl) phosphate (TBEP) (median 16 ng/L, max. 93 ng/L), TCEP (median 10 ng/L, maximum 41 ng/L) and tris(dichloropropyl) phosphate (TDCPP) (median 11 ng/L, maximum 28 ng/L) (Table 1). The concentration profiles for TCPP, TiBP and TCEP are shown in Fig. 7. Slightly higher concentrations of TCPP and TCEP are mainly found in the upstream part of the Danube in Germany. Further downstream, concentrations of OPCs are relatively stable, indicating constant inputs via wastewater treatment plants (WWTPs). Elevated concentrations are found in the Timok, Iskar and Arges tributaries. The concentrations of OPCs detected are in good agreement with the findings of other monitoring studies (Andresen et al., 2004, Martinez-Carballo et al., 2007, Regnery and Püttmann, 2010, Van der Veen and de Boer, 2012, Wolschke et al., 2015). Martinez-Carballo et al. (2007), who sampled the Danube in Vienna in 2005, found lower concentrations of TCPP (38 ng/L against 74 ng/L found by us in 2013), but higher concentrations of TCEP (20 ng/L in 2005, compared with 11 ng/L in 2013) and TiBP (65 ng/L in 2005, compared with 15 ng/L in 2013), which indicates increased use of TCPP, and decreased use of TCEP and TiBP; this reflects the observed changes in the production and use of OPCs reported by Wolschke et al. (2015).

### 3.7 Environmental quality standards

EU Directive 2008/105/EU (EU, 2008) sets EQS for priority substances and certain other pollutants in order to limit the concentrations of certain chemical substances that pose a significant risk to the environment or to human health in surface waters in the European Union (EU) with the aim of achieving ‘good’ surface water chemical status. Table 1 gives the (annual average) EQS limits for the substances investigated in surface water. Cybutryne, terbutryn and PFOS are new priority substances under the WFD, and therefore official European-wide EQS values are specified in Directive 2013/39/EU (EU, 2013). In addition, for diclofenac, an EQS of 0.1 μg/L was proposed in 2011 (European Commission (2012) 876 final). In several European countries, national EQS for other ‘river basin-specific pollutants’ have been derived, e.g. for Switzerland by the Swiss Centre for Applied Ecotoxicology (http://www.ecotoxcentre.ch/expert-service/quality-standards/).

Among the compounds analysed in JDS3, the EU water EQS was exceeded only for PFOS (along the whole Danube River) which has a very low EQS of 0.65 ng/L. The only national water EQS (see Table 1) to be exceeded was that for diclofenac (0.1 μg/L) in the Arges tributary (255 ng/L).

### 3.8 PFOS in fish liver and SPM

PFOS was also analysed in liver of the Danube white bream because of its high bioaccumulation potential. The analytical monitoring results for JDS3 and JDS2 samples are shown in Table 2. Four fish liver samples from JDS3 (2013) and three fish liver samples (plus one fillet sample) from JDS2 (2007) were analysed. The biota EQS for PFOS of 9.1 μg/kg (EU, 2013) was exceeded in all cases, also for the filet sample from JDS2 (26 μg/kg). However, because the number of samples analysed was low, no clear temporal or local trend in PFOS contamination could be identified. It should be noted that the goal of setting an EQS for PFOS is to prevent excessive intake of PFOS by humans who consume fish; therefore, fish muscle or fillet should be analysed for EQS compliance checking. However, most biota monitoring studies so far have focused either on liver, as a target organ for PFOS accumulation, or on blood or whole-body homogenates (Berger et al., 2009, Houde et al., 2006, Houde et al., 2011). Analyses of 10 harbour seal organs showed that perfluorinated compounds (PFCs) tend to accumulate primarily in blood (38% of the total PFC burden) and liver (36%). Concentrations are lower in muscle (13%), lung (8%), kidney (2%), blubber (2%), heart (1%), brain (1%), thymus (< 0.01%) and thyroid (< 0.01%) (Ahrens et al., 2009).

**Table 2:** Monitoring results for PFOS in fish liver (and one filet) from 2013 and 2007 N, number of samples (2013) = 4; (2007) = 4; unit: μg/kg; LOD = 0.2 μg/kg; LOQ = 0.5 μg/kg.

Code	Location name	Year 2013	Year 2007
JDS2	Kelheim – gauging station (DE)	529	329
JDS20	Szob (HU)	329	
JDS27	Hercegszanto (HU)	284	26 (filet)
JDS63	Siret Tributary (RO)		864
JDS65	Reni (RO/UA)	109	
JDS68	Sf.Gheorghe - Sf.Gheorghe arm (RO)		1007

**Fig. 8:**
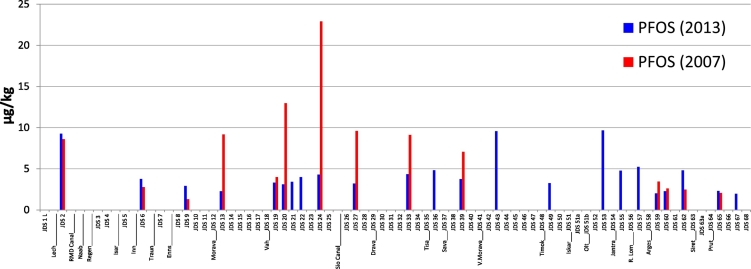
PFOS concentration profile in SPM along the Danube River and tributaries.

PFOS was also analysed in SPM; the analytical results for JDS3 and JDS2 are given in Fig. 8 and Table SI11. In agreement with the water analysis, pollution levels were generally similar in 2013 (median 3.75 μg/kg) and 2007 (median 3.99 μg/kg) although in the middle of the river (JDS20–JDS39) PFOS levels in SPM were lower in 2013 (Fig. 8).

### 3.9 Mass discharge and chemical emissions to the Black Sea

The mass discharge load emissions (in tons/year) of the most relevant chemicals for JDS2 are presented in Loos et al. (2010). During JDS2, the flow of the Danube was measured only at some points, with the following results: ~ 1180 m^3^/s in Austria; ~ 1400 m^3^/s in Hungary after Budapest; ~ 2460 m^3^/s in Serbia (downstream of the Tisa); in Romania ~ 3200 m^3^/s downstream of the Velika Morava tributary, ~ 4830 m^3^/s downstream of the Timok tributary and ~ 6420 m^3^/s downstream of the Arges tributary. River flow during JDS3 was similar (3203 m^3^/s at JDS42) up to the Iron Gate Dam (JDS44), located downstream of the Velika Morava tributary. At this dam, the water was ponded in August 2013, so that the flow of the Danube River downstream of the dam remained relatively constant at around 3000 m^3^/s up to its delta at JDS65. For JDS3, the mass load fluxes for the organic contaminants could be calculated along the whole river (reported for the most relevant substances in Table SI12) using the water discharge data collected during the survey. The contribution of the downstream tributaries is small. The mass flux calculations for the most relevant chemicals, given in Table 3, show that the maximum loads were not found at the river mouth at JDS65, because of the reduced river flow downstream of the Iron Gate Dam. The highest loads were calculated for TCPP (48.5 tons/year), 1-H-benzotriazole (29.8 tons/year) and the methylbenzotriazoles (10.5 tons/year).

**Table 3:** Mass loads of the most relevant chemicals in the Danube River in (t/year).

Substance	Max. mass load	Mass load at JDS65
1-H-Benzotriazole	29.8 (JDS27)	24.1
Methylbenzotriazoles	10.5 (JDS24)	5.4
Carbamazepine	3.2 (JDS33)	2.2
Carbamazepine-diOH	4.7 (JDS46)	3.5
Sulfamethoxazole	1.8 (JDS46)	1.4
PFOA	1.9 (JDS13)	0.47
PFOS	1.3 (JDS20)	0.54
TiBP	2.7 (JDS33)	0.64
TCPP	48.5 (JDS32)	27.8
TCEP	2.8 (JDS43)	0.88

## 4 Conclusions

The Danube continues to show signs of degradation downstream of major cities and in a number of important tributaries as a result of insufficient or non-existent treatment of wastewater. Treated and untreated wastewater released from WWTPs is the main source of organic contaminants found in the Danube River and its tributaries. The concentrations of the selected emerging organic micropollutants analysed were relatively low in the Danube River, and in most cases higher in the tributaries. The highest concentrations and loads were found for benzotriazole corrosion inhibitors and organophosphorus flame retardants. While the concentrations of the analysed WFD priority substances and national river basin-specific pollutants are generally below their EQS values, concentrations of PFOS in water and fish exceed the EQS by a factor of around 10. Concentrations of PFOA have more or less halved since 2007, but it is still an important pollutant in the Danube River Basin. PFOS concentrations decreased only slightly from 2007 to 2013, which suggests the emission pathways of PFOS and PFOA are different. Concentrations of pharmaceuticals (carbamazepine and sulfamethoxazole) are relatively constant over time; similar levels were found in 2007 and 2013. A further reduction in nutrients and organic pollution is needed.

## Appendix A Supplementary data

Supplementary material Image 2
