# Supplementary material for: Analysis of emerging organic contaminants in water, fish and suspended particulate matter (SPM) in the Joint Danube Survey using solid-phase extraction followed by UHPLC-MS-MS and GC–MS analysis
Source: Sci Total Environ. 2017 Dec 31;607-608:1201–12. doi: 10.1016/j.scitotenv.2017.07.039 (PMC5600344; doi:10.1016/j.scitotenv.2017.07.039)
Supplement: Supplementary file 1 — Supplementary material [file mmc1.docx]

**Table SI1**

**Target organic compounds analysed by SPE-UHPLC-MS-MS and SPE-GC-MS.** New WFD priority substances and “watch list” substance (diclofenac) in blue font.

| **Analyte** | **CAS No.** | **Log K_OW_** | **Description / use** |
| --- | --- | --- | --- |
| 1-H-Benzotriazole | 95-14-7 | 1.44 | Corrosion inhibitor |
| 5-Methyl-1H-benzotriazole | 136-85-6 | 1.7 | Corrosion inhibitor |
| Carbamazepine (CBZ) | 298-46-4 | 2.15-2.45 | Mood-stabilizing drug |
| 10,11-Dihydro-10,11-dihydroxy-carbamazepine (CBZ-metabolite; CBZ-diOH) | 58955-93-4 | - 0.21 | Degradation product of carbamazepine |
| Diclofenac | 15307-86-5 | 4.51 | Non-steroidal anti-inflammatory drug |
| Sulfamethoxazole | 723-46-6 | 0.48 | Sulfonamide antibiotic |
| 2,4-D (2,4-dichlorophenoxyacetic acid) | 94-75-7 | 2.83 | Herbicide |
| MCPA (2-methyl-4-chlorophenoxyacetic acid) | 94-74-6 | 2.73 | Herbicide |
| Metolachlor | 51218-45-2 | 3.13 | Herbicide |
| Cybutryne (irgarol) | 28159-98-0 | 3.38 | Biocide, fungicide |
| Terbutryn | 886-50-0 | 3.49 | Herbicide and biocide |
| DEET (N,N-diethyl-m-toluamide) | 134-62-3 | 2.0 | Insecticide |
| PFBS (perfluorobutane sulfonic acid) | 375-73-5 | n.a. | Perfluoroalkyl substance |
| PFHxA (perfluorohexanoic acid) | 307-24-4 | n.a. | Perfluoroalkyl substance |
| PFHpA (perfluoroheptanoic acid) | 375-85-9 | n.a. | Perfluoroalkyl substance |
| PFOA (perfluorooctanoic acid) | 335-67-1 | n.a. | Perfluoroalkyl substance |
| PFNA (perfluorononanoic acid) | 375-95-1 | n.a. | Perfluoroalkyl substance |
| PFOS (perfluorooctane sulfonic acid) | 1763-23-1 | n.a. | Perfluoroalkyl substance |
| Tri-n-propyl phosphate (TnPP) | 513-08-6 | n.a. | Flame retardant |
| Tris(isobutyl) phosphate (TiBP) | 126-71-6 | 3.6 | Flame retardant |
| Tris(n-butyl) phosphate (TnBP) | 126-73-8 | 4.0 | Flame retardant |
| Tris(1-chloro-2-propyl) phosphate (TCPP) | 13674-84-5 | 2.59 | Flame retardant |
| Tris(2-chloroethyl) phosphate (TCEP) | 115-96-8 | 1.44 | Flame retardant |
| Tris(2-butoxyethyl) phosphate (TBEP) | 78-51-3 | 3.65 | Flame retardant |
| Tris(1,3-dichloropropyl) phosphate  Tris(2,3-dichloropropyl) phosphate (TDCPP) | 13674-87-8  78-43-3 | 3.8 | Flame retardant |
| Triphenyl phosphate (TPhP) | 115-86-6 | 4.6 | Flame retardant |
| 2-Ethylhexyl diphenyl phosphate (EHDP) | 1241-94-7 | n.a. | Flame retardant |
| Tris(methylphenyl) phosphate (TMPP), Tritolyl phosphate, Tricresylphosphate | 1330-78-5 | 5.9 | Flame retardant |
| Tris(3,5-dimethylphenyl) phosphate (T35DMPP) | 25653-16-1 | n.a. | Flame retardant |

**Table SI2**

**Internal surrogate standards used for isotope dilution analysis**

| 1-H-Benzotriazole d_4_ |
| --- |
| Carbamazepine d_10_ |
| ^13^C_6_-Diclofenac |
| ^13^C_6_-Sulfamethoxazole |
| 2,4-D (2,4-dichlorophenoxyacetic acid) d_6_ |
| MCPA (2-methyl-4-chlorophenoxyacetic acid) d_3_ |
| Metolachlor d_6_ |
| Cybutryne d_9_ |
| Terbutryn d_5_ |
| DEET (N,N-diethyl-m-toluamide) d_6_ |
| ^13^C_2_-PFHxA (perfluorohexanoic acid) |
| ^18^O_2_-PFHxS (perfluorohexane sulfonic acid) |
| ^13^C_4_-PFOA (perfluorooctanoic acid) |
| ^13^C_5_-PFNA (perfluorononanoic acid) |
| ^13^C_4_-PFOS (perfluorooctane sulfonic acid) |

**OPCs**

| Tri-n-propyl phosphate-d_21_ (TnPP-d_21_) |
| --- |
| Tris(butyl) phosphate-d_27_ (TnBP-d_27_) |
| Triphenyl phosphate-d_15_ (TPhP-d_15_) |
| Tris(2-butoxy(^13^C_2_)-ethyl) phosphate (M6TBEP) |
| Tris(1,3-dichloro-2-propyl) phosphate-d_15_ (TDCPP-d_15_) |
| Tris(3,5-dimethylphenyl) phosphate-d_9_ (T35DMPP-d_9_) |
| Tris(2-chloroethyl) phosphate-d_12_ (TCEP-d_12_) |

**Table SI3**

**SPE recoveries of the target UHPLC-MS-MS compounds**

| **Analyte** | **Recovery (%)** |
| --- | --- |
| 1H-Benzotriazole | 31 |
| 5-Methyl-1H-benzotriazole | 72 |
| Carbamazepine (CBZ) | 79 |
| 10,11-Dihydro-10,11-dihydroxy-carbamazepine (CBZ-diOH) | 78 |
| Diclofenac | 83 |
| Sulfamethoxazole | 86 |
| 2,4-D (2,4-dichlorophenoxyacetic acid) | 31 |
| MCPA (2-methyl-4-chlorophenoxyacetic acid) | 41 |
| Metolachlor | 99 |
| Cybutryne (irgarol) | 111 |
| Terbutryn | 85 |
| DEET (N,N-diethyl-m-toluamide) | 93 |
| PFBS (perfluorobutane sulfonic acid) | 58 |
| PFHxA (perfluorohexanoic acid) | 43 |
| PFHpA (perfluoroheptanoic acid) | 170 |
| PFOA (perfluorooctanoic acid) | 96 |
| PFNA (perfluorononanoic acid) | 75 |
| PFOS (perfluorooctane sulfonic acid) | 43 |

**Table SI4**

**Percentage concentration decrease (or increase) between the two analyses after 55 and 173 days (“118 day variation”)**

Unit: (%)

|  | **JDS 11** | **JDS 12** | **JDS 16** |
| --- | --- | --- | --- |
| 1H-Benzotriazole | -26 | -57 | -45 |
| 5-Methyl-1H-benzotriazole | 210 | 38 | 118 |
| Carbamazepine | 11 | -21 | -11 |
| Carbamazepine-diOH | -4 | -12 | -22 |
| Diclofenac | -50 | -87 | -76 |
| Sulfamethoxazole | -11 | -46 | -21 |
| 2,4-D | -16 | -30 | -23 |
| MCPA | 4 | -12 | 31 |
| Metolachlor | -27 | -64 | -39 |
| Cybutryne | > 75 | n.d. | n.d. |
| Terbutryn | -21 | -27 | -28 |
| DEET | -95 | -95 | -94 |
| PFBS | -58 | n.d. | n.d. |
| PFHxA | -37 | -7 | -41 |
| PFHpA | > 75 | n.d. | n.d. |
| PFOA | -32 | 19 | -44 |
| PFNA | -71 | > 75 | n.d. |
| PFOS | -18 | 1 | 0 |

**Table SI5**

**UHPLC-MS-MS MRM analytical conditions**

RT: Retention time; Q1: Precursor ion; Q3: Product ion; DP: Declustering Potential; EP: Entrance Potential; CE: Collision Energy; CXP: Collision Cell Entrance Potential.

| **Analyte** | **RT (min)** | **Q1** | **Q3** | **DP (V)** | **EP (V)** | **CE(V)** | **CXP (V)** |
| --- | --- | --- | --- | --- | --- | --- | --- |
| 1H-Benzotriazole | 3.1 | 120 | 65 | 209 | 10 | 29 | 13 |
| 1H-Benzotriazole 1 | 3.1 | 120 | 92 | 209 | 10 | 24 | 13 |
| Benzotriazole d_4_ | 3.1 | 124 | 69 | 56 | 10 | 35 | 13 |
| Sulfamethoxazole | 3.3 | 254 | 156 | 150 | 10 | 22 | 13 |
| Sulfamethoxazole 1 | 3.3 | 254 | 92 | 150 | 10 | 38 | 13 |
| Sulfamethoxazole ^13^C_6_ | 3.3 | 260 | 98 | 70 | 10 | 36 | 13 |
| 10,11-Dihydro-10,11-dihydroxycarbamazepine | 3.6 | 271 | 180 | 80 | 10 | 47 | 13 |
| 10,11-Dihydro-10,11-dihydroxycarbamazepine 1 | 3.6 | 271 | 210 | 80 | 10 | 19 | 13 |
| 10,11-Dihydro-10,11-dihydroxycarbamazepine 2 | 3.6 | 271 | 253 | 80 | 10 | 10 | 13 |
| Carbamazepine | 4.2 | 237 | 194 | 250 | 10 | 28 | 13 |
| Carbamazepine 1 | 4.2 | 237 | 165 | 250 | 10 | 60 | 13 |
| Carbamazepine d_10_ | 4.2 | 247 | 204 | 234 | 10 | 31 | 13 |
| PFHxA | 4.2 | 313 | 269 | -107 | -10 | -12 | -11 |
| PFHxA 1 | 4.2 | 313 | 119 | -107 | -10 | -28 | -11 |
| PFHxA ^13^C_2_ | 4.2 | 315 | 270 | -60 | -10 | -13 | -11 |
| PFBS | 4.4 | 299 | 80 | -260 | -10 | -66 | -11 |
| PFBS 1 | 4.4 | 299 | 99 | -260 | -10 | -39 | -11 |
| 2,4-D | 4.5 | 219 | 161 | -130 | -10 | -24 | -11 |
| 2,4-D 1 | 4.5 | 219 | 125 | -130 | -10 | -38 | -11 |
| 2,4-D ^13^C_6_ | 4.5 | 225 | 167 | -68 | -10 | -19 | -11 |
| DEET | 4.5 | 192 | 91 | 244 | 10 | 41 | 13 |
| DEET 1 | 4.5 | 192 | 119 | 244 | 10 | 24 | 13 |
| DEET d_6_ | 4.5 | 198 | 91 | 80 | 10 | 42 | 13 |
| MCPA | 4.5 | 199 | 141 | -147 | -10 | -21 | -11 |
| MCPA 1 | 4.5 | 199 | 105 | -147 | -10 | -40 | -11 |
| PFHpA | 4.7 | 363 | 319 | -116 | -10 | -14 | -11 |
| PFHpA 1 | 4.7 | 363 | 169 | -116 | -10 | -24 | -11 |
| PFHxS ^18^O_2_ | 4.9 | 403 | 84 | -297 | -10 | -125 | -11 |
| Terbutryn | 5.0 | 242 | 186 | 255 | 10 | 25 | 13 |
| Terbutryn 1 | 5.0 | 242 | 91 | 255 | 10 | 36 | 13 |
| Terbutryn d_5_ | 5.0 | 247 | 191 | 228 | 10 | 27 | 13 |
| Terbutryn d_5_ 1 | 5.0 | 247 | 91 | 228 | 10 | 36 | 13 |
| Cybutryne | 5.1 | 254 | 198 | 261 | 10 | 26 | 13 |
| Cybutryne 1 | 5.1 | 254 | 74 | 261 | 10 | 30 | 13 |
| Cybutryne d_9_ | 5.1 | 263 | 199 | 269 | 10 | 27 | 13 |
| Cybutryne d_9_ 1 | 5.1 | 263 | 74 | 269 | 10 | 61 | 13 |
| PFOA | 5.1 | 413 | 369 | -122 | -10 | -16 | -11 |
| PFOA 1 | 5.1 | 413 | 169 | -122 | -10 | -26 | -11 |
| PFOA ^13^C_4_ | 5.1 | 417 | 372 | -119 | -10 | -15 | -11 |
| Metolachlor | 5.3 | 284 | 252 | 200 | 10 | 22 | 13 |
| Metolachlor 1 | 5.3 | 284 | 176 | 200 | 10 | 35 | 13 |
| Diclofenac | 5.4 | 294 | 250 | -42 | -10 | -16 | -11 |
| Diclofenac 1 | 5.4 | 294 | 214 | -42 | -10 | -29 | -11 |
| Diclofenac ^13^C_6_ | 5.4 | 300 | 256 | -173 | -10 | -15 | -11 |
| PFNA | 5.4 | 463 | 419 | -122 | -10 | -19 | -11 |
| PFNA 1 | 5.4 | 463 | 219 | -122 | -10 | -25 | -11 |
| PFNA 2 | 5.4 | 463 | 169 | -122 | -10 | -27 | -11 |
| PFNA ^13^C_5_ | 5.4 | 468 | 423 | -57 | -10 | -16 | -11 |
| PFOS | 5.5 | 499 | 80 | -260 | -10 | -97 | -11 |
| PFOS 1 | 5.5 | 499 | 99 | -260 | -10 | -83 | -11 |
| PFOS ^13^C_4_ | 5.5 | 503 | 80 | -276 | -10 | -104 | -11 |

**Table SI6**

**Linearity**

Linearity was studied in the concentration range 0.2-100 ng/L (real water concentration). 1-L Milli-Q water blank samples spiked only with labelled internal standards and five 1-L Milli-Q water samples spiked with native analytes mixture (i.e. spiking level 0.2, 1, 2, 40, 100 ng/L or 0.1, 0.5, 1, 20 and 50 ng/L for perfluorinated compounds) were extracted and analyzed on five different days. The linearity of the whole analytical method including extraction and UHPLC-MS-MS analysis was estimated by calculating the regression coefficients (R^2^) and the results are reported in the following table:

| **Analyte** | **Mean Slope** | **Mean Intercept** | **Mean R^2^** |
| --- | --- | --- | --- |
| 1H-Benzotriazole | 0.008277485 | 0.002844723 | 0.9963 |
| 5-Methyl-1H-benzotriazole | 0.009997261 | -0.008096145 | 0.9950 |
| Carbamazepine (CBZ) | 0.033835417 | 0.033284745 | 0.9980 |
| CBZ-diOH | 0.013589018 | 0.076998096 | 0.9313 |
| Diclofenac | 0.078652144 | -0.03422926 | 0.9983 |
| Sulfamethoxazole | 0.009985115 | -0.006783511 | 0.9972 |
| 2,4-D | 0.004337204 | -0.002305979 | 0.9978 |
| MCPA | 0.006100681 | 0.003224974 | 0.9994 |
| Metolachlor | 0.011156292 | 0.005042873 | 0.9987 |
| Cybutryne | 0.029478022 | -0.008624483 | 0.9975 |
| Terbutryn | 0.003230371 | 0.004339579 | 0.9990 |
| DEET | 0.003938568 | 0.013389058 | 0.9996 |
| PFBS | 0.110966722 | 0.092353137 | 0.9990 |
| PFHxA | 0.034176131 | 0.021219538 | 0.9981 |
| PFHpA | 0.135187722 | 0.152803289 | 0.9996 |
| PFOA | 0.152376255 | 0.386062868 | 0.9973 |
| PFNA | 0.026402697 | 0.02309762 | 0.9927 |
| PFOS | 0.095412676 | 0.147261619 | 0.9862 |

The mean R^2^ values (calculated over five calibration curves) were ≥ 0.99 for all analytes, except PFOS with an R^2^ value of 0.986.

**Table SI6-2**

Linearity for the organophosphate compounds analysed by GC-MS was studied in the real water concentration range between 10-500 ng/L on 8 different days. R^2^ values are reported in the table below.

| Date / Analyte | 23/9/13 | 24/9/13 | 25/9/13 | 30/9/13 | 16/10/13 | 7/11/13 | 18/11/13 | 3/3/14 | **Average** |
| --- | --- | --- | --- | --- | --- | --- | --- | --- | --- |
| **TnPP** | 0.9980 | 0.9991 | 0.9982 | 0.9996 | 0.9993 | 0.9997 | 0.9994 | 0.9998 | 1.00 |
| **TiBP** | 0.9953 | 0.9998 | 0.9999 | 1.0000 | 0.9994 | 0.9997 | 0.9995 | 0.9999 | 1.00 |
| **TnBP** | 0.9999 | 0.9997 | 0.9995 | 0.9998 | 0.9991 | 1.0000 | 0.9993 | 1.0000 | 1.00 |
| **TCPP** | 0.9970 | 0.9982 | 0.9970 | 0.9987 | 0.9985 | 0.9992 | 0.9991 | 0.9998 | 1.00 |
| **TCEP** | 0.9998 | 0.9998 | 0.9996 | 0.9998 | 0.9996 | 1.0000 | 0.9997 | 1.0000 | 1.00 |
| **TBEP** | 0.9996 | 0.9999 | 0.9997 | 1.0000 | 0.9997 | 0.9997 | 0.9999 | 0.9888 | 1.00 |
| **TDCPP** | 0.9999 | 1.0000 | 0.9992 | 0.9996 | 0.9992 | 1.0000 | 0.9998 | 0.9938 | 1.00 |
| **TPhP** | 0.9999 | 0.9998 | 0.9996 | 0.9999 | 0.9995 | 1.0000 | 0.9997 | 0.9998 | 1.00 |
| **EHDP** | 0.9664 | 0.9879 | 0.9994 | 0.9914 | 0.9996 | 0.8524 | 0.9988 | 0.9999 | 0.97 |
| **TMPP** | 0.9689 | 0.9355 | 0.9923 | 0.9901 | 0.9971 | 0.8879 | 0.9984 | 0.9646 | 0.97 |
| **T35DMPP** | 0.9981 | 0.9996 | 0.9987 | 0.9991 | 0.9996 | 0.9387 | 0.9999 | 0.9663 | 0.99 |

**Table SI7**

**Repeatability, day-to-day variation, intermediate precision**

For repeatability, intermediate precision and day-to-day variation estimation, quality control samples of two concentration levels were tested on different days. For each sample three replicate injections were made. Using one-way ANOVA, the following results were obtained as shown in the table below; the RSDs are given in (%).

| **Analyte** | **Lower concentration level** | | | **Higher concentration level** | | |
| --- | --- | --- | --- | --- | --- | --- |
|  | Repeat-ability (%) | Day-to-day variation (%) | Inter-mediate precision (%) | Repeat-ability (%) | Day-to-day variation (%) | Inter-mediate precision (%) |
| 1H-Benzotriazole | 5.2 | 17.3 | 18.0 | 4.6 | 18.3 | 18.9 |
| 5-Methyl-1H-benzotriazole | 5.0 | 2.9 | 5.8 | 4.1 | 11.5 | 12.2 |
| Carbamazepine (CBZ) | 7.2 | 4.2 | 8.3 | 4.0 | 2.3 | 4.6 |
| CBZ-diOH | 6.3 | 22.7 | 23.5 | 21.2 | 19.3 | 28.6 |
| Diclofenac | 21.3 | 12.0 | 24.4 | 6.9 | 3.6 | 7.8 |
| Sulfamethoxazole | 1.7 | 1.0 | 1.9 | 2.0 | 0.8 | 2.1 |
| 2,4-D | 4.0 | 9.4 | 10.2 | 5.6 | n.a. | n.a. |
| MCPA | 4.6 | 2.6 | 5.3 | 4.9 | 2.5 | 5.5 |
| Metolachlor | 8.4 | 4.8 | 9.7 | 6.7 | 2.9 | 7.3 |
| Cybutryne | 18.4 | 10.6 | 21.3 | 16.1 | 11.2 | 19.3 |
| Terbutryn | 12.0 | 7.0 | 13.9 | 3.8 | 2.2 | 4.4 |
| DEET | 31.2 | 18.0 | 36.0 | 7.9 | 9.8 | 12.6 |
| PFBS | n.a. | n.a. | n.a. | 5.2 | n.a. | n.a. |
| PFHxA | 21.5 | 14.4 | 25.9 | 12.6 | 7.7 | 14.8 |
| PFHpA | n.a. | n.a. | n.a. | 14.5 | 10.2 | 17.7 |
| PFOA | n.a. | n.a. | n.a. | 6.4 | 3.5 | 7.3 |
| PFNA | 22.0 | 12.3 | 25.2 | 20.2 | 13.4 | 24.2 |
| PFOS | n.a. | n.a. | n.a. | 19.6 | 12.7 | 23.4 |

**Table SI7-2**

For the analysis of the OCPs results of replicate analysis of control samples were used for repeatability (reproducibility) evaluation presented as relative standard deviations (RSDs) or coefficients of variance (CV). The results reported in the table below should not exceed the value of 30 %.

|  | **RSD or coefficient of variance (CV) (%)** | |
| --- | --- | --- |
|  | **Low concentration**  **(30 ng/L)** | **High concentration**  **(300 ng/L)** |
| T*n*PP | 4 | 11 |
| T*i*BP | 3 | 15 |
| T*n*BP | 2 | 10 |
| TCPP | 2 | 21 |
| TCEP | 6 | 10 |
| TBEP | 6 | 12 |
| TDCPP | 3 | 8 |
| TPhP | 2 | 7 |
| EHDP | 4 | 17 |
| TMPP | 3 | 16 |
| T35DMPP | 4 | 13 |

**Table SI8**

**Limits of detection (LODs) and quantification (LOQs)**

| **Analyte** | **LOD (ng/L)** | **LOQ (ng/L)** |
| --- | --- | --- |
| 1H-Benzotriazole | 0.31 | 0.66 |
| 5-Methyl-1H-benzotriazole | 0.26 | 0.53 |
| Carbamazepine | 0.07 | 0.15 |
| 10,11-Dihydro-10,11-dihydroxy-carbamazepine | 0.13 | 0.30 |
| Diclofenac | 0.34 | 0.86 |
| Sulfamethoxazole | 0.05 | 0.10 |
| 2,4-D | 0.09 | 0.22 |
| MCPA | 0.06 | 0.15 |
| Metolachlor | 0.66 | 1.73 |
| Cybutryne (Irgarol) | 0.07 | 0.18 |
| Terbutryn | 0.33 | 0.64 |
| DEET | 0.73 | 1.93 |
| PFBS | 0.19 | 0.55 |
| PFHxA | 0.5 | 1.10 |
| PFHpA | 1.5 | 3.20 |
| PFOA | 0.46 | 1.07 |
| PFNA | 0.29 | 0.66 |
| PFOS | 0.53 | 1.09 |
| T*n*PP | 0.21 | 0.70 |
| T*i*BP | 0.15 | 0.51 |
| T*n*BP | 0.09 | 0.30 |
| TCPP | 1.29 | 4.30 |
| TEHP | 0.035 | 0.12 |
| TBEP | 1.51 | 5.03 |
| TDCPP | 0.85 | 2.83 |
| TCEP | 0.29 | 0.96 |
| TPhP | 0.16 | 0.52 |
| EHDP | 0.084 | 0.28 |
| TMPP | 0.24 | 0.79 |
| T35DMPP | 1.03 | 3.44 |
| T2*i*PPP | 12.0 | 39.9 |

**Table SI9**

**Equipment and conditions of the GC-MS system**

| GC | Agilent 6890 N |  |  |  |
| --- | --- | --- | --- | --- |
| Column: | RXI-17SIL MS |  |  |  |
| Nominal length: | 60 m |  |  |  |
| Nominal Diameter: | 250 µm |  |  |  |
| Nominal film thick­ness: | 0.25 µm |  |  |  |
| Gas Type | Helium |  |  |  |
| Mode: | constant flow |  |  |  |
| Initial flow: | 1.5 mL/min |  |  |  |
| Oven: |  |  |  |  |
| Initial Temperature: | 80 ͦC |  |  |  |
| Initial Time: | 1 min |  |  |  |
| Ramps: | # | Rate | Final Temp | Final Time |
|  | 1 | 30 ͦC/min | 180 ͦC | 0 min |
|  | 2 | 10 ͦC/min | 300 ͦC | 15 min |
| Run Time | 31.33 min |  |  |  |

| Front Inlet (CIS4) |  |  |  |  |
| --- | --- | --- | --- | --- |
| Mode | Splitless |  |  |  |
| Initial Temperature | 250 ͦC |  |  |  |
| Pressure | 186 kPa |  |  |  |
| Purge Flow | 100 mL/min |  |  |  |
| Purge Time | 1 min |  |  |  |
| Total Flow | 104.2 mL/min |  |  |  |

| Gerstel CIS 4 |  |  |  |  |
| --- | --- | --- | --- | --- |
| Initial Temperature | 80 ͦC |  |  |  |
| Equilibration Time | 0.05 min |  |  |  |
| Initial Time | 0.10 min |  |  |  |
| Rate | 10 ͦC/sec |  |  |  |
| Final Temp | 280 ͦC |  |  |  |
| Hold Time | 10 min |  |  |  |

| MS | Agilent 5973 Mass Selective Detector |  |  |  |
| --- | --- | --- | --- | --- |
| Mode | EI |  |  |  |
| MS Quad | 150 ͦC |  |  |  |
| MS Source | 230 ͦC |  |  |  |

**Table SI10**

**GC-MS SIM parameters for OPC analytes**

| Analyte/IS Abbreviation | m/z (target) *amu* | m/z (qualifier) *amu* |
| --- | --- | --- |
| TnPP | 99 | 141 |
| TnPP-d21 | 103 | 151 |
| TiBP | 99 | 155 |
| TnBP | 99 | 155 |
| TnBP-d27 | 103 | 167 |
| TCPP | 277 | 279 |
| TEHP | 99 | 113 |
| TCEP | 249 | 251 |
| TCEP-d12 | 263 | 264 |
| TBEP | 227 | 299 |
| M6TBEP | 303 | 259 |
| TDCPP | 379 | 381 |
| TDCPP-d15 | 394 | 197 |
| TPhP | 325 | 326 |
| TPhP-d15 | 339 | 341 |
| EHDP | 251 | 362 |
| TMPP | 367 | 368 |
| T35DMPP | 410 | 411 |
| T35DMPP-d9 | 420 | 421 |
| T2iPPP | 335 | 293 |

**Table SI11**

**Monitoring results for PFOS in SPM from 2013 and 2007**

N, number of samples (2013) = 23; (2007) = 15; unit: µg/kg; LOD = 0.2 µg/kg; LOQ = 0.5 µg/kg.

|  | **Location name** | **Year 2013** | **Year 2007** |
| --- | --- | --- | --- |
| **JDS2** | Kelheim – gauging station (DE) | 9.27 | 8.60 |
| **JDS6** | Jochenstein (DE) | 3.77 | 2.78 |
| **JDS9** | Klosterneuburg (AT) | 2.92 | 1.31 |
| **JDS13** | Bratislava (SK) | 2.28 | 9.18 |
| **JDS19** | Iza/Szony (HU) | 3.32 | 3.99 |
| **JDS20** | Szob (HU) | 3.11 | 12.98 |
| **JDS21** | Budapest upstream - Megyeri Bridge (HU) | 3.42 |  |
| **JDS22** | Budapest downstream - M0 bridge (HU) | 3.99 |  |
| **JDS24** | Dunafoldvar (HU) | 4.30 | 22.91 |
| **JDS27** | Hercegszanto (HU) | 3.22 | 9.60 |
| **JDS33** | Downstream Novi-Sad (RS) | 4.35 | 9.12 |
| **JDS36** | Downstream Tisa / Upstream Sava (Belegis) (RS) | 4.83 |  |
| **JDS39** | Downstream Pancevo (RS) | 3.75 | 7.06 |
| **JDS43** | Banatska Palanka / Bazias (RS/RO) | 9.57 |  |
| **JDS49** | Pristol / Novo Selo Harbour (RO/BG) | 3.26 |  |
| **JDS53** | Downstream Zimnicea / Svishtov (RO/BG) | 9.67 |  |
| **JDS55** | Downstream Jantra (RO/BG) | 4.77 |  |
| **JDS57** | Downstream Ruse / Giurgiu (RO/BG) | 5.24 |  |
| **JDS59** | Downstream Arges, Oltenita (RO/BG) | 2.02 | 3.45 |
| **JDS60** | Chiciu / Silistra (RO/BG) | 2.28 | 2.61 |
| **JDS62** | Braila (RO) | 4.81 | 2.47 |
| **JDS65** | Reni (RO/UA) | 2.32 | 2.07 |
| **JDS67** | Sulina - Sulina arm (RO) | 1.96 | < LOQ |
|  | **Median** | **3.75** | **3.99** |

**Table SI12**

**Mass loads of the most relevant chemicals along the Danube River in t/year.**

1-H-Benzotriazole = 1BT; Methylbenzotriazoles = MBT; Carbamazepine = CMZ; CMZ-diOH = CMZO; Sulfamethoxazole = SMZ

|  |  | Flow | 1BT | MBT | CMZ | CMZO | SMZ | PFOA | PFOS | TiBP | TCPP | TCEP |
| --- | --- | --- | --- | --- | --- | --- | --- | --- | --- | --- | --- | --- |
| JDS3 code | rkm | (m3/h) | [t/year] | [t/year] | [t/year] | [t/year] | [t/year] | [t/year] | [t/year] | [t/year] | [t/year] | [t/year] |
|  |  |  |  |  |  |  |  |  |  |  |  |  |
| JDS2 | 2415 | 230 | 3.35 | 0.70 | 0.23 | 0.49 | 0.26 | 0.02 | 0.17 | 0.35 | 0.69 | 0.13 |
| JDS3 | 2354 | 245 | 2.49 | 0.54 | 0.23 | 0.34 | 0.26 | 0.02 | 0.08 | 0.18 | 1.28 | 0.18 |
| JDS4 | 2285 | 288 | 3.12 | 0.52 | 0.31 | 0.40 | 0.39 | 0.04 | 0.12 | 0.22 | 1.65 | 0.20 |
| JDS5 | 2258 | 353 | 5.32 | 1.02 | 0.37 | 0.81 | 0.48 | 0.05 | 0.17 | 0.22 | 1.30 | 0.19 |
| JDS6 | 2205 | 887 | 7.74 | 1.37 | 0.78 | 1.40 | 0.58 | 0.26 | 0.45 | 0.48 | 1.79 | 0.19 |
| JDS7 | 2120 | 981 | 8.11 | 0.61 | 0.76 | 0.89 | 0.75 | 0.22 | 0.17 | 0.45 | 2.77 | 0.33 |
| JDS8 | 2008 | 1158 | 7.72 | 1.07 | 0.63 | 0.93 | 0.67 | 0.23 | 0.20 | 0.47 | 8.69 | 1.02 |
| JDS9 | 1942 | 1122 | 11.04 | 1.83 | 0.80 | 1.95 | 1.10 | 0.70 | 0.35 | 0.30 | 1.98 | 0.34 |
| JDS10 | 1895 | 1212 | 11.18 | 2.10 | 0.71 | 1.16 | 0.81 | 0.23 | 0.22 | 0.83 | 3.50 | 0.45 |
| JDS11 | 1881 | 1978 | 20.07 | 3.81 | 1.45 | 3.36 | 1.56 | 1.13 | 0.43 | 1.02 | 3.63 | 0.50 |
| Morava  JDS12 | 1880 | 31 | 0.369 | 0.143 | 0.046 | 0.112 | 0.046 | 0.005 | 0.010 | 0.012 | 0.130 | 0.012 |
| JDS13 | 1869 | 1881 | 12.55 | 2.28 | 0.93 | 2.10 | 0.89 | 1.90 | 0.53 | 0.82 | 2.69 | 0.24 |
| JDS15 | 1806 | 1414 | 15.50 | 3.30 | 1.13 | 2.76 | 1.12 | 0.96 | 0.00 | 0.68 | 2.64 | 0.42 |
| JDS16 | 1794 | 49 | 0.84 | 0.21 | 0.09 | 0.23 | 0.05 | 0.03 | 0.01 | 0.03 | 0.15 | 0.02 |
| JDS17 | 1790 | 1463 | 16.80 | 4.50 | 1.23 | 2.70 | 1.34 | 0.37 | 0.41 | 0.80 | 2.67 | 0.46 |
| Vah  JDS18 | 1766 | 99 | 4.840 | 0.320 | 0.096 | 0.290 | 0.070 | 0.013 | 0.030 | 0.154 | 0.270 | 0.038 |
| JDS19 | 1761 | 1527 | 17.44 | 4.76 | 1.56 | 2.35 | 1.16 | 1.20 | 0.98 | 0.77 | 2.52 | 0.42 |
| JDS20 | 1707 | 1521 | 26.51 | 4.97 | 1.70 | 0.99 | 1.22 | 0.54 | 1.26 | 0.87 | 4.77 | 0.50 |
| JDS21 | 1660 | 1470 | 20.85 | 4.07 | 1.24 | 3.01 | 1.21 | 1.20 | 0.27 | 0.78 | 3.40 | 0.45 |
| JDS22 | 1632 | 1483 | 22.09 | 4.34 | 1.64 | 3.75 | 1.27 | 1.71 | 0.11 | 0.83 | 3.61 | 0.41 |
| JDS24 | 1560 | 1781 | 26.35 | 10.46 | 1.97 | 4.47 | 1.31 | 1.01 | 0.46 | 0.86 | 4.47 | 0.52 |
| JDS25 | 1533 | 1922 | 23.56 | 3.27 | 1.91 | 2.59 | 1.17 | 0.43 | 0.47 | 1.04 | 6.76 | 0.63 |
| JDS26 | 1481 | 1871 | 22.49 | 3.53 | 1.46 | 2.45 | 1.02 | 0.33 | 0.42 | 1.07 | 7.27 | 0.66 |
| JDS27 | 1434 | 1898 | 29.83 | 7.32 | 1.92 | 3.44 | 1.46 | 0.99 | 0.50 | 0.87 | 4.72 | 0.54 |
| JDS28 | 1384 | 1922 | 21.98 | 2.93 | 2.83 | 3.63 | 1.20 | 1.07 | 0.50 | 1.00 | 5.59 | 0.75 |
| Drava  JDS29 | 1379 | 493 | 2.004 | 0.517 | 0.419 | 0.582 | 0.058 | 0.182 | 0.078 | 0.323 | 0.563 | 0.052 |
| JDS30 | 1367 | 2485 | 23.56 | 6.81 | 2.30 | 4.09 | 1.25 | 1.52 | 0.58 | 1.36 | 5.76 | 0.60 |
| JDS31 | 1300 | 2529 | 23.93 | 2.88 | 1.66 | 2.83 | 0.97 | 0.41 | 0.50 | 1.49 | 6.67 | 0.77 |
| JDS32 | 1262 | 2642 | 28.77 | 3.57 | 2.76 | 4.13 | 1.37 | 0.51 | 0.63 | 0.80 | 48.53 | 1.02 |
| JDS33 | 1252 | 2816 | 26.48 | 6.30 | 3.15 | 4.51 | 1.16 | 1.08 | 1.18 | 2.67 | 7.25 | 0.72 |
| JDS34 | 1216 | 2750 | 24.24 | 6.71 | 2.66 | 3.93 | 1.29 | 1.18 | 0.85 | 1.99 | 7.01 | 0.71 |
| Tisa  JDS35 | 1215 | 252 | 0.483 | 0.639 | 0.270 | 0.664 | 0.141 | 0.065 | 0.000 | 0.102 | 0.573 | 0.080 |
| JDS36 | 1200 | 2986 | 26.30 | 3.52 | 2.11 | 3.67 | 1.04 | 1.13 | 0.49 | 2.16 | 6.18 | 0.65 |
| Sava  JDS37 | 1170 | 385 | 0.763 | 0.215 | 0.108 | 0.294 | 0.197 | 0.007 | 0.044 | 0.057 | 0.339 | 0.029 |
| JDS38 | 1159 | 3050 | 20.33 | 2.61 | 2.08 | 3.24 | 1.20 | 0.32 | 0.36 | 1.52 | 5.52 | 0.56 |
| JDS39 | 1151 | 2995 | 19.96 | 4.05 | 1.89 | 3.66 | 1.22 | 0.41 | 0.45 | 1.44 | 5.60 | 0.55 |
| JDS40 | 1107 | 2869 | 20.66 | 3.88 | 2.08 | 3.93 | 1.18 | 0.34 | 0.51 | 1.62 | 5.90 | 0.53 |
| V. Morava  JDS41 | 1103 | 46 | 0.196 | 0.095 | 0.039 | 0.161 | 0.074 | 0.007 | 0.007 | 0.034 | 0.122 | 0.018 |
| JDS42 | 1097 | 3203 | 22.87 | 4.11 | 2.40 | 4.09 | 1.25 | 0.42 | 0.50 | 1.50 | 6.16 | 0.64 |
| JDS43 | 1071 | 2680 | 21.21 | 4.25 | 2.14 | 3.53 | 1.24 | 0.37 | 0.50 | 1.29 | 10.31 | 2.81 |
| JDS44 | 1040 | 3000 | 23.92 | 3.42 | 2.32 | 4.10 | 1.08 | 0.35 | 0.52 | 1.46 | 9.73 | 1.38 |
| JDS45 | 954 | 3146 | 24.52 | 6.73 | 2.46 | 4.36 | 1.62 | 0.42 | 0.61 | 1.43 | 11.12 | 1.04 |
| JDS46 | 926 | 3299 | 25.71 | 7.69 | 2.53 | 4.69 | 1.78 | 0.43 | 0.63 | 1.59 | 12.13 | 0.99 |
| JDS47 | 851 | 2547 | 16.52 | 4.04 | 1.49 | 3.29 | 1.33 | 0.31 | 0.33 | 1.51 | 5.40 | 0.40 |
| JDS49 | 834 | 2528 | 20.46 | 7.44 | 1.79 | 3.42 | 1.40 | 0.41 | 0.44 | 1.48 | 5.87 | 0.72 |
| JDS52 | 602 | 2604 | 22.00 | 9.91 | 1.61 | 3.25 | 1.23 | 0.42 | 0.44 | 2.03 | 5.66 | 0.51 |
| JDS53 | 550 | 2620 | 20.58 | 5.98 | 1.52 | 2.81 | 1.14 | 0.36 | 0.39 | 1.78 | 9.47 | 0.88 |
| Jantra  JDS54 | 537 | 14 | 0.010 | 0.009 | 0.003 | 0.014 | 0.008 | 0.001 | 0.004 | 0.007 | 0.033 | 0.007 |
| JDS55 | 532 | 2672 | 19.56 | 6.18 | 1.60 | 3.02 | 1.06 | 0.27 | 0.41 | 1.76 | 7.28 | 0.67 |
| JDS59 | 429 | 2816 | 25.13 | 7.41 | 2.09 | 3.36 | 1.61 | 0.50 | 0.56 | 2.04 | 9.53 | 0.62 |
| JDS60 | 378 | 2792 | 24.09 | 7.08 | 2.02 | 3.63 | 1.74 | 0.39 | 0.48 | 2.24 | 8.99 | 0.71 |
| JDS61 | 235 | 2851 | 19.51 | 1.69 | 2.16 | 4.51 | 1.44 | 0.34 | 0.51 | 1.68 | 11.10 | 0.78 |
| JDS62 | 167 | 2901 | 21.92 | 6.48 | 1.80 | 3.91 | 1.37 | 0.47 | 0.46 | 2.05 | 11.13 | 0.44 |
| Siret  JDS63 | 154 | 80 | 0.067 | 0.117 | 0.009 | 0.032 | 0.022 | 0.003 | 0.011 | 0.054 | 0.242 | 0.021 |
| Prut  JDS64 | 135 | 61 | 0.014 | 0.045 | 0.010 | 0.031 | 0.026 | 0.003 | 0.000 | 0.014 | 0.067 | 0.019 |
| **JDS65** | **130** | 2998 | **24.08** | **5.42** | **2.15** | **3.49** | **1.43** | **0.47** | **0.54** | **0.64** | **27.81** | **0.88** |
| JDS66 | 18 | 1353 | 10.30 | 2.22 | 0.99 | 1.54 | 0.70 | 0.18 | 0.30 | 1.16 | 3.35 | 0.34 |
| JDS67 | 26 | 738 | 6.02 | 1.80 | 0.66 | 1.08 | 0.49 | 0.15 | 0.16 | 0.63 | 2.79 | 0.50 |
| JDS68 | 107 | 1002 | 5.53 | 0.84 | 0.54 | 1.09 | 0.46 | 0.10 | 0.16 | 0.59 | 1.71 | 0.17 |
|  |  |  |  |  |  |  |  |  |  |  |  |  |
| Median |  |  | 19.96 | 3.42 | 1.52 | 2.81 | 1.14 | 0.37 | 0.42 | 0.86 | 4.72 | 0.50 |
| **Max.** |  |  | **29.83** | **10.46** | **3.15** | **4.69** | **1.78** | **1.90** | **1.26** | **2.67** | **48.53** | **2.81** |

**Figure SI1**

**Ratio CBZ / CBZ-diOH along the Danube River**

**Figure SI2**

**DEET concentration profile along the Danube River and tributaries.** Tributaries in blue font.

**Figure SI3**

**2,4-D concentration profile along the Danube River and tributaries for the years 2013 and 2007**
